# GSP-2, a polysaccharide extracted from *Ganoderma sinense*, is a novel toll-like receptor 4 agonist

**DOI:** 10.1371/journal.pone.0221636

**Published:** 2019-08-23

**Authors:** Kai-Sheng Liu, Cheng Zhang, Hong-Liang Dong, Kai-Kai Li, Quan-Bin Han, Yong Wan, Rui Chen, Fang Yang, Hai-Li Li, Chun-Hay Ko, Xiao-Qiang Han

**Affiliations:** 1 The First Affiliated Hospital of Southern University of Science and Technology, The Second Clinical Medical College of Jinan University, Shenzhen People’s Hospital, Shenzhen, Guangdong, China; 2 Institute of Chinese Medicine, The Chinese University of Hong Kong, Shatin, New Territories, Hong Kong SAR, China; 3 Institute of Biology and Medical Sciences, Soochow University, Suzhou, Jiangsu, China; 4 College of Food Science and Technology, Huazhong Agricultural University, Wuhan, Hubei, China; 5 School of Chinese Medicine, Hong Kong Baptist University, Kowloon, Hong Kong SAR, China; 6 Shenzhen Research Institute, The Chinese University of Hong Kong, Shenzhen, Guangdong, China; Universidade Federal do Rio de Janeiro, BRAZIL

## Abstract

*Ganoderma sinense* is a Chinese unique medicinal fungus that has been used in folk medicine for thousands of years. Polysaccharides are considered to be biologically active ingredients due to their immune-modulating functions. Previously we found that GSP-2, a new polysaccharide isolated from *Ganoderma sinense*, exerts an immunomodulatory effect in human peripheral blood mononuclear cells but the underlying mechanism is unclear. The present study aimed to investigate how GSP-2 triggers immunologic responses and the implicated signaling pathways. GSP-2 effects were investigated both in a macrophagic cell line, RAW264.7, and in primary macrophages. Moreover, the molecular basis of GSP-2 recognition by immune cells, and the consequent activation of signaling cascades, were explored by employing recombinant human HEK293-TLR-Blue clones, individually overexpressing various Toll-like receptors. GSP-2 dose-dependently induced the overexpression of Toll-like receptor 4 (TLR4) but did not affect the expression of other TLRs. Moreover, GSP-2 induced TNFα secretion in primary macrophages from wild-type, but not TLR4-knockout mice. In addition, GSP-2 upregulated TLR4 protein expression and activated the MAPK pathway in RAW246.7 macrophages. Finally, GSP-2 induced the production of the cytokines TNFα, IL1β, and IL6. Our data demonstrated that GSP-2 was specifically recognized by TLR4, promoting cytokine secretion and immune modulation in macrophages.

## Introduction

*Ganoderma sinense* (GS) is a Chinese unique medicinal fungus that has a long history of use in traditional Chinese medicine [1]. This fungus was recorded in the Chinese pharmacopoeia 2010 as Ling Zhi, together with its relative, *G*. *Lucidum* (LG) [2]. In recent years, GS has attracted much attention in the area of immune therapy for its beneficial adjuvant effects in the treatment of some serious and chronic diseases such as cancer [3–6]. The small molecular ingredients contained in GS is lower than its sister spice LG. Macromolecular polysaccharides from GS fruiting bodies are widely considered to be involved in the immune-modulatory activity of this fungus [3, 4]. Due to the complex structure and the high molecular weight of the polysaccharide fractions, their detailed chemical structure and mechanism of action are still unknown.

GSP-2 is a water-soluble protein-bound glucan that has been isolated from GS [7]. GSP-2 contains a backbone composed of (1→4)—and (1→6)-linked β-D-glucopyranosyl residues, bearing the side chains of (1→3)—and terminal-linked β-D-glucopyranosyl residues at O-3 position of (1→6)-linked β-D-glucopyranosyl residues, as well as trace amounts of galactose and mannose residues [7]. Interestingly, GSP-2 is a B cell immunomodulator while it does not affect mouse splenic T cells *in vitro*. GSP-2 strongly stimulates cytokine secretion from PBMCs and derived dendritic cells. The underlying mechanism, as well as the identity of the putative GSP-2 receptor on B cells, have not been identified.

Toll-like receptors (TLRs) are a class of proteins playing an important role in innate immunity [8–10]. They are divided into multiple subtypes, and the transmembrane subtype is implicated in the recognition of polysaccharides [11, 12]. TLRs are usually expressed by sentinel cells such as macrophages and dendritic cells, which recognize structurally conserved molecules derived from microbes [13–15]. The TLR class includes 13 members (TLR1-13). TLR12 and TLR13 have not been found in humans [16–18]. TLRs are important for the recognition of polysaccharides and some other natural polymers [19]. Ando *et al* found that polysaccharides from safflower activate the transcription factor, NF-kappa B, via TLR4 [20]. Wei *et el* found that TLR4 mediates signaling events induced by the Astragalus polysaccharide, RAP [18]. While the structural complexity of polysaccharides has been finally clarified, the details of their interaction with TLRs are still elusive.

The present study is the first to investigate the mechanism by which GSP-2 triggers downstream signaling pathways and modulates immune responses. Our results demonstrated the existence of a specific GSP-2-TLR4 immunomodulatory axis and identified the implicated signaling cascades.

## Materials and methods

### Cell culture and reagents

RAW264.7 macrophages (ATCC, Rockville, MD, USA) (4×10^5^ cell/ well) were seeded in 24-well plates with DMEM medium (Gibco, USA), 10% fetal bovine serum (FBS) at 37 °C in 5% CO_2_ overnight. The extraction and preparation of GSP-2 has been reported before [7], and the same batch of GSP-2 was used in this study. PMB was purchased from Sigma (Deisenhofen, Germany). Anti-Toll-like receptor 4, anti-β-actin, anti-JNK, anti-p38, anti-ERK, anti- phospho-p38, anti-phospho-ERK, and anti-phospho-JNK were purchased from Cell Signaling Technology (Danvers, MA, USA).

### Endotoxin detection

Detection and quantitative evaluation of endotoxin contamination in GSP-2 were conducted by using rFC-based assays following the manufacturer’s instructions (PyroGene^®^ Recombinant Factor C Endotoxin Detection System, LONZA, USA). Briefly, a 100 μl mixture containing endotoxin standards and GSP-2 was dispensed into 96-well microplates and pre-incubated for 10 minutes at 37°C. Next, 100 μl of working reagent, consisting of fluorogenic substrate, assay buffer, and rFC enzyme solution in a 5:4:1 ratio, were added as appropriate. The optical density (OD) was measured both immediately and after a 1-h incubation at 37 °C by using a fluorescence microplate reader (BMG, Germany).

### Nitric oxide production

The culture supernatant of RAW264.7 macrophages was added to Griess Reagent in a 1:1 ratio in a 96-well plate, followed by incubation in the dark for 10 min. The OD value was then measured at a 540 nm wavelength. A NaNO_2_ solution was used to plot a nitrite standard curve after Griess treatment.

### TLR screening

TLR screening was performed using a panel of HEK293-TLR-Blue cell lines, each of which was engineered to express a single specific mouse TLR (mTLR2, 3, 4, 5, 7, 8, 9, and 13, respectively) and an NF-κB–inducible secreted embryonic alkaline phosphatase (SEAP) reporter protein. TLR ligand screening was performed by InvivoGen company. Briefly, GSP-2 and controls were incubated for 18 h with the recombinant HEK293-TLR-Blue cell lines, with or without PMB. The following known TLR agonists were used as positive controls: PAM2 (100 ng/ml) for mTLR2; poly (I:C) (100 ng/ml) for mTLR3; Escherichia coli K12 LPS (1 μg/ml) for TLR4; flagellin (1 μg/ml) for mTLR5; R848 (10 μg/ml) for mTLR7, CL075 + poly dt (10 μg/ml)for mTLR8, ODN 2006 (1 μg/ml) for mTLR9, and ORN Sa19 (1 μg/ml) for mTLR13. A recombinant HEK-293 cell line (mTLR-), which did not express any TLR gene was used as a negative control. An increase in SEAP activity was indicative of cell activation, which was assessed by measuring the absorbance at 630 nm.

### Preparation of mouse peritoneal macrophages

C57BL/6, TLR2 or TLR4 knockout mice were treated with 3% thioglycolate three days before they were sacrificed by cervical amputation. Peritoneal lavage was performed to harvest peritoneal macrophages with ice-cold Ca^2+^- and Mg^2+^-free PBS. Collected cells were centrifuged and resuspended in ice-cold PBS. All animal experiments were approved by the Ethics Committee of Shenzhen People’s Hospital (No.LL-KY-2019142).

### Activation of macrophages in vitro

Freshly harvested macrophages from C57BL/6, TLR2 or TLR4 knockout mice were treated with various concentrations of GSP-2 (from 12.5 to 100 μg/ml) in 96-well plates for 24 h at 37 °C. The culture supernatant was then harvested and assayed for TNF-α using ELISA kits (Biolegend, USA) following the manufacturer’s instructions.

### Western blot

RAW264.7 cells were lysed in RIPA buffer for 30 min on ice. After centrifugation at 12000 rpm for 15 min at 4 °C, protein concentration was measured in the supernatants by the BCA method. Next, 40 μg of protein were separated by SDS–PAGE and then transferred onto PVDF membranes (GE, USA). The membranes were blocked with 5% milk for 1 h and incubated with the primary antibody overnight at 4 °C. The membranes were washed three times with Tris Buffered Saline-Tween 20 (TBST), and then incubated with the corresponding secondary antibody for 1 h at room temperature. Finally, chemiluminescent detection (Bio-Rad, USA) was performed.

### PCR array

RAW264.7 cells were cultured in 6 well plates and then stimulated with GSP-2 (50 μg/ml) in the presence of PMB (10 μg/ml) for 18 h. Control cells were treated with LPS (0.35 ng/ml) in the presence of PMB (10 μg/ml). The mRNA was isolated from RAW264.7 using an RNeasy Mini Kit (Qiagen, Germany). Each group was repeated three times. mRNA isolated from the three repeated experiments (in the same group) was mixed together. Next, PCR array was performed with the Toll-Like Receptor Signaling Pathway RT^2^ Profiler PCR Array System (PAMM-18Z, Qiagen, Germany). 0.8 μg of total mRNA from each group was transcribed into cDNA using an RT^2^ First Strand Kit. cDNA was added to RT^2^ SYBR Green Mastermix. The aliquot mix was added into RT^2^ Profiler PCR Arrays. Real-time PCR array was performed according to the manufacturer’s protocol on a BioRad CFX96 PCR System. The mRNA level of 84 genes was analyzed. Multiple housekeeping genes were used for normalization. The normalized data were analyzed by comparing 2^−ΔΔCt^ values. Fold changes were calculated relative to control group.

### Quantitative real time RT-PCR (qRT-PCR)

The mRNA was isolated from RAW264.7 cells by using an RNeasy Mini Kit (Qiagen, Hilden, Germany). Selected genes were amplified and quantified by one-step real-time PCR (CFX 96, Bio-rad, USA) using Quantifast SYBR Green RT-PCR kit (Qiagen, Germany), following the manufacturer’s protocol. Data were normalized to endogenous GAPDH and analyzed by comparing 2^−ΔΔCt^ values. Fold changes were calculated relative to untreated RAW264.7 cells. The sequences of used primers are listed in S1 Table.

### Statistical analysis

Statistical analyses and significance were measured by one-way ANOVA. Multiple comparison between the groups was performed using Dunnett’s Multiple Comparison Test. Data were expressed as mean ± SD of three repeats. *p < 0.05; **p < 0.01; ***p < 0.001 compared to control.

## Results

### GSP-2 activates nitric oxide production

**Fig 1:**
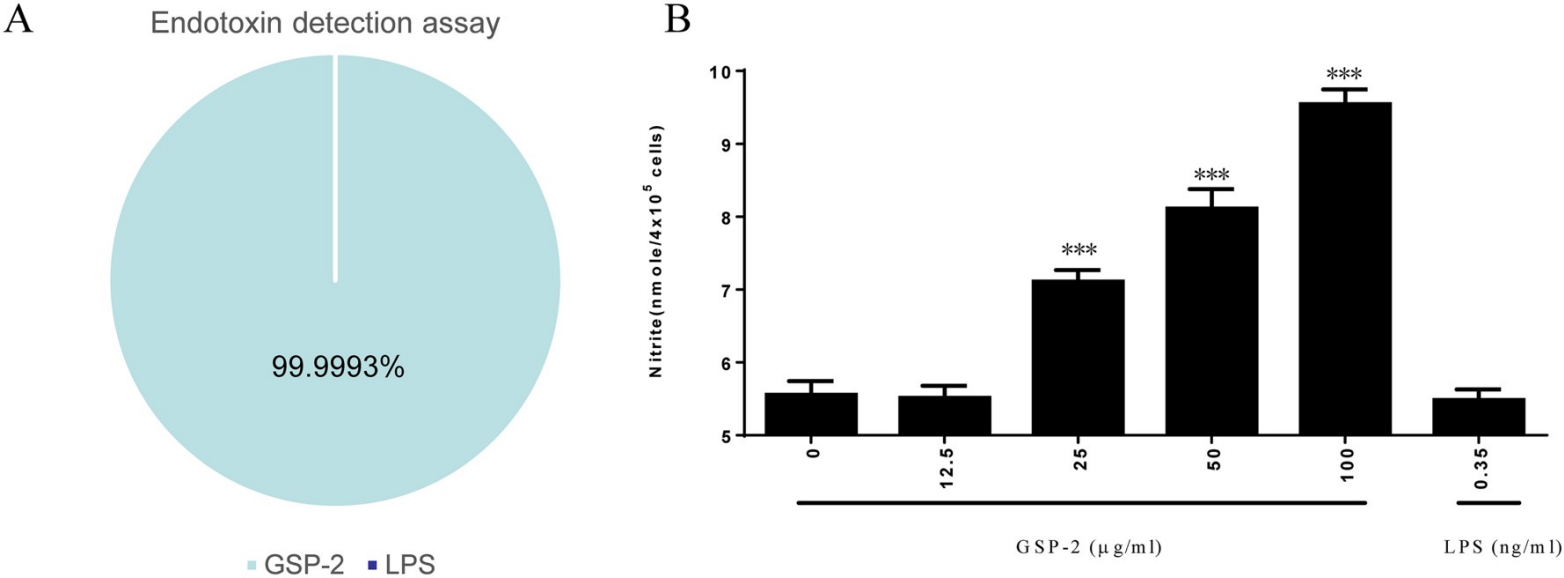
Endotoxin detection assay for GSP-2 and effect of GSP-2 on NO release in RAW264.7 cells. **(A)** Quantification of LPS in the GSP-2 preparation. An endotoxin detection assay was performed to evaluate the LPS concentration in GSP-2. **(B)** RAW264.7 cells (4×10^5^ cells/ml) were treated with GSP-2 or LPS in Polymyxin B (PMB, 10 μg/ml) for 18 h in DMEM. The nitrite levels in the supernatants were determined using the Griess reagent. All values are expressed as mean ± SD of three repeats. *p < 0.05; **p < 0.01; ***p < 0.001 compared to control. Statistical significance was determined using the one-way ANOVA.

Polysaccharides isolated from *Ganoderma sinense* may be contaminated by LPS, which could induce a signaling cascade leading to the activation of NF-κB and the production of pro-inflammatory cytokines typical of innate immunity [21, 22]. Therefore, LPS was quantified by an endotoxin detection assay in the GSP-2 fraction. In a 50 μg/ml GSP-2 solution, LPS concentration was 0.35±0.017 ng/ml, corresponding to 0.0007±0.00003% LPS (Fig 1A).

RAW264.7 cells (4×10^5^ cells/ml) were treated with GSP-2 or LPS in polymyxin B (PMB, 10 μg/ml) for 18 h in Dulbecco’s Modified Eagle’s medium (DMEM), and then the nitrite levels were determined in the supernatant. The results showed that GSP-2 induced nitric oxide production in a dose-dependent manner in RAW264.7 cells and that LPS can be blocked by PMB (Fig 1B), indicating that the effect of LPS can be excluded in the following studies.

### GSP-2 interacts with TLR4 directly and stimulates its expression

**Fig 2:**
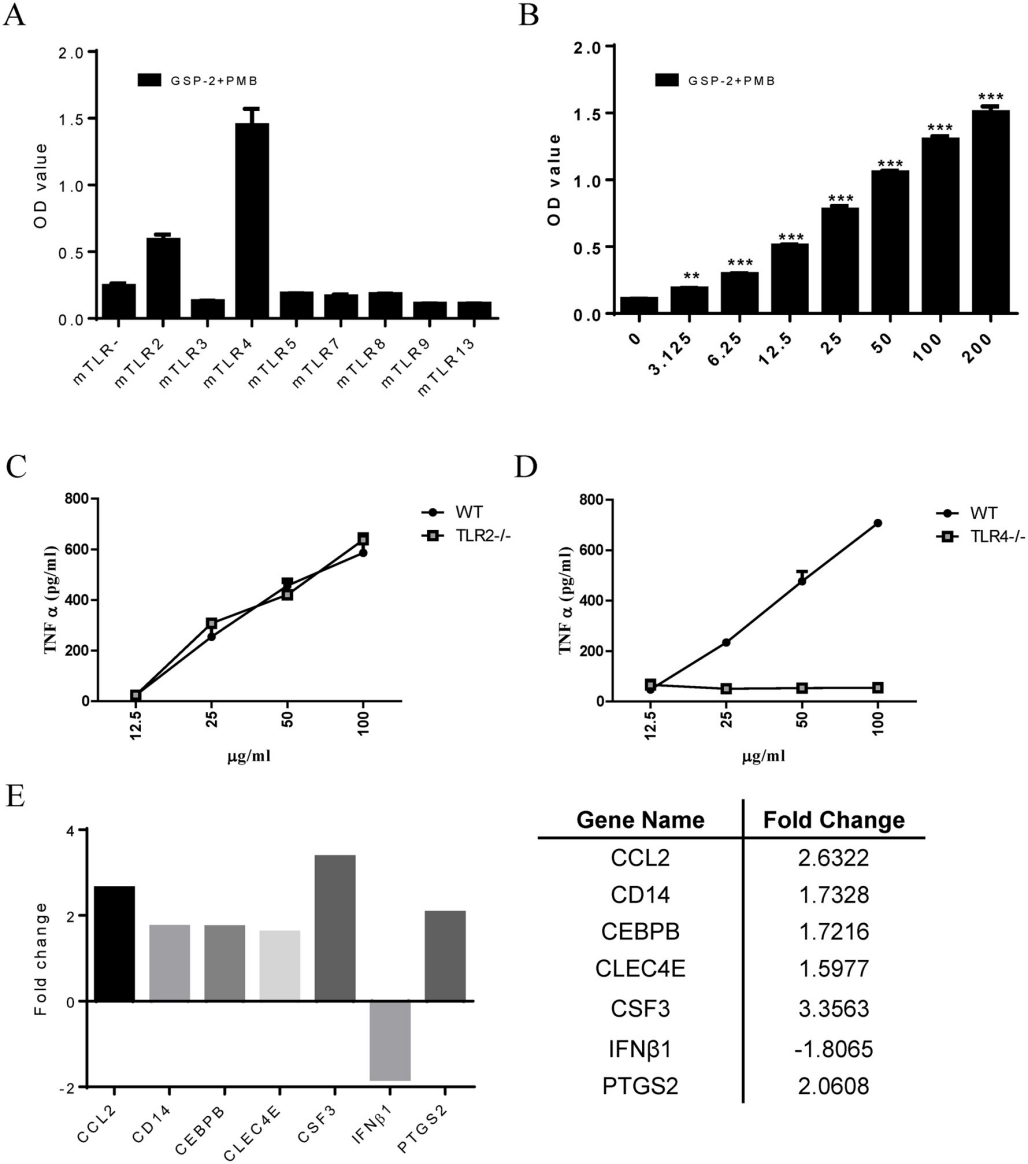
Effect of GSP-2 on the activation of Toll-like receptors in engineered HEK293 cell lines. **(A)** Identification of GSP-2-interacting TLRs. mTLR2, 3, 4, 5, 7, 8, 9, and 13 are engineered HEK-293 cell lines that stably overexpress murine TLR2, 3, 4, 5, 7, 8, 9, and 13 gene, respectively, while mTLR- does not express any TLR gene. The cell clones also co-expressed an NF-κB–inducible secreted embryonic alkaline phosphatase (SEAP) reporter gene. Secreted SEAP levels were detected with QUANTI-Blue medium and quantified by measuring the optical density. The cells were incubated with GSP-2 (50 μg/ml) in the presence of polymyxin B (PMB, 10 μg/ml) for 18 h. Values are expressed as mean ± SD of three repeats. **(B)** GSP-2 dose-response curve in the mTLR4 cell line. mTLR4 cells were incubated with the indicated GSP-2 concentrations in the presence of PMB (10 μg/ml) for 18 h. Control cells were incubated with normal medium. Values are expressed as mean ± SD of three repeats. *p < 0.05; **p < 0.01; ***p < 0.001 compared to control. **(C and D)** Effect of GSP-2 on TNFα secretion in mouse primary macrophages. Peritoneal macrophages harvested from TLR2 or TLR4 C57BL/6 knockout mice were incubated with various concentration of GSP-2 for 24 h. The culture supernatant was assayed for TNFα by ELISA. Wild-type (WT) macrophages were harvested from normal C57BL/6 mice. Values are expressed as mean ± SD of three repeats. **(E)** Effect of GSP-2 on the expression of TLR genes. RAW264.7 cells were stimulated with GSP-2 (50 μg/ml) in the presence of PMB (10 μg/ml) for 18 h. Control cells were treated with LPS (0.35 ng/ml) in the presence of PMB (10 μg/ml). The differential expression of key genes of TLR signaling pathways were analyzed by PCR array. Fold change values greater than ±1.5 are shown. Fold change values for each of the differentially expressed genes are shown in the right panel.

To identify the specific receptor of GSP-2, we selectively evaluated the possible role of different TLRs utilizing the HEK-Blue TLR clones, which stably co-express single mouse TLRs (TLR2 to TLR13) and an NF-κB-inducible SEAP reporter gene. SEAP release into the medium was used as a measure of TLR activation. Our previous studies showed that 50 μg/ml GSP-2 can efficiently induce immune responses, therefore we used this concentration for the assay. PMB (10 μg/ml) was used to block NF-κB activation induced by contaminating LPS. GSP-2, in the presence of PMB, specifically and very strongly activated mTLR4-expressing cells, and weakly activated mTLR2 cells (Fig 2A). Notably, NF-κB was not activated in the other mTLR cell clones or in the absence of TLR expression (mTLR-). As expected, mTLR clones could be activated by the respective known TLR agonists. These results indicated that, among TLR members, TLR4 directly interacted with GSP-2 on the cell surface and, therefore, was the most likely candidate GSP-2 receptor.

To investigate GSP-2 dose-response effects on TLR4 activation, we incubated mTLR4 cells with increasing GSP-2 concentrations in the presence of PMB (10 μg/ml), to rule out possible effects due to LPS contamination. A 18-h treatment with GSP-2 significantly stimulated mTLR4 cells in a dose-dependent manner (Fig 2B).

Macrophages are a crucial component of the innate immune system and promote local and systemic inflammation [23]. Macrophages are the major producers of TNFα, which, in turn, induces the expression of several other immunoregulatory mediators in innate immunity [24]. To confirm the TLR screening results, we examined GSP-2-induced TNFα secretion in primary macrophages from knockout TLR4 and TLR2 C57BL/6 mice. GSP-2 promoted TNFα secretion both in wild-type and in TLR2-/- cells (Fig 2C), whereas it was ineffective in TLR4-/- primary macrophages (Fig 2D). This suggested that TLR4, but not TLR2, plays a pivotal role in GSP-2-mediated macrophage activation.

To investigate signaling events induced by GSP-2 through TLR4, we examined gene expression in RAW264.7 stimulated with GSP-2 (50 μg/ml) in the presence of PMB for 18 h, using the mouse toll-like receptor signaling pathway PCR array analysis. To block the possible effects of trace LPS amounts, RAW246.7 treated with 0.35 ng/ml LPS (equal to the LPS concentration found in 50 μg/ml GSP-2, as above established), were used as control, in the presence of PMB as an LPS inhibitor. The mRNA level of 84 genes was analyzed and listed in S2 Table. GSP-2 upregulated pathogen-specific response genes (including PTGS2, CD14, and CLEC4E) and genes involved in cytokine effector functions (including CCL2, CEBPB, and CSF3), while IFNβ1 was suppressed (Fig 2E).

### GSP-2 activates TLR4 and the MAPK pathway in RAW264.7 macrophages

**Fig 3:**
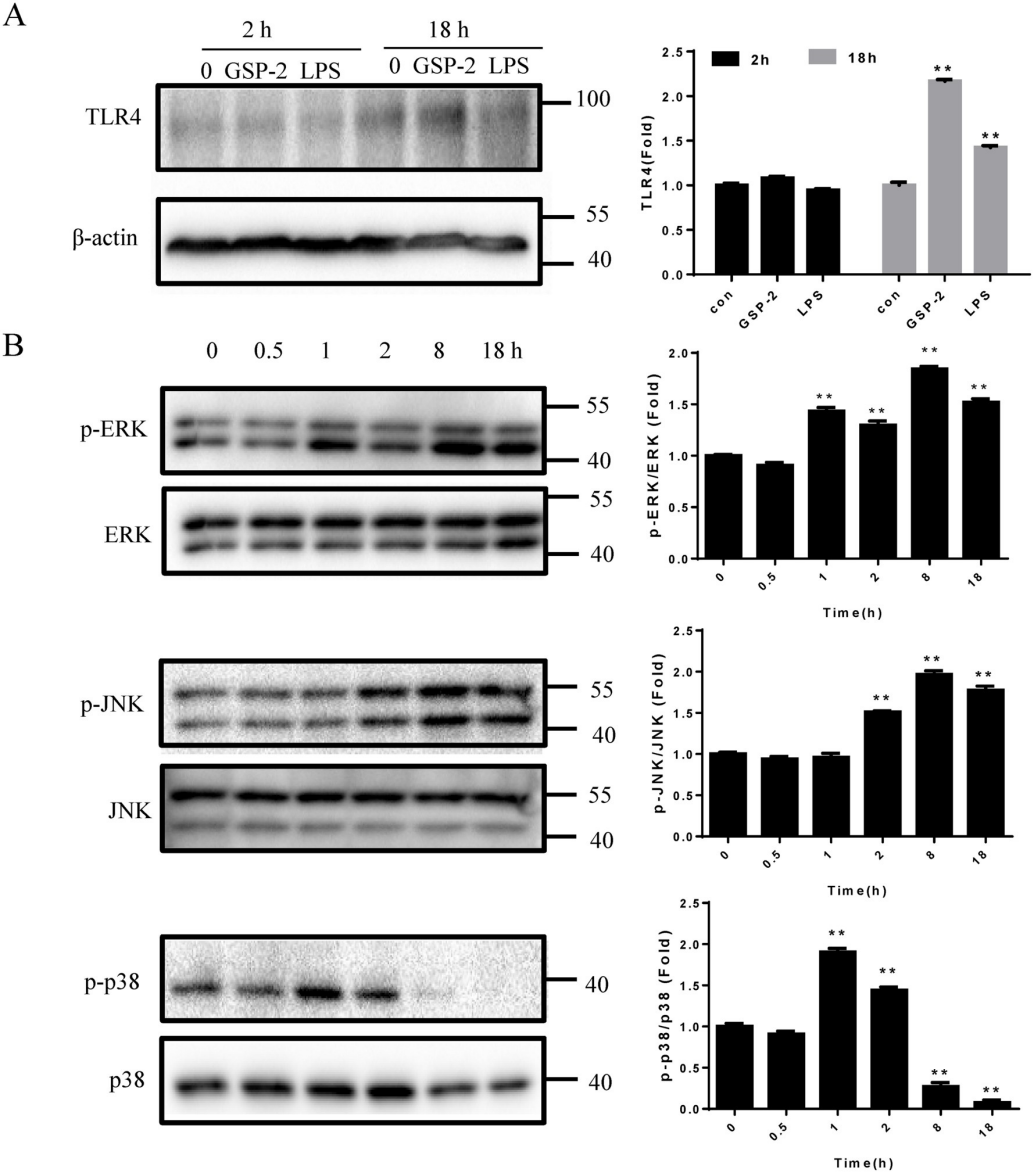
Effect of GSP-2 on TLR4 protein expression and the MAPK pathway in RAW264.7 cells. **(A)** RAW264.7 cells were stimulated with GSP-2 (50 μg/ml) or LPS (1 μg/ml) with PMB (10 μg/ml) for the indicated times, then the proteins were extracted and TLR4 was analyzed by western blotting. β-actin was used as a loading control. **(B)** RAW264.7 cells were stimulated with GSP-2 (50 μg/ml) with PMB for the indicated times, then the proteins were extracted and ERK, JNK, p38, and their phosphorylated forms were analyzed by western blotting. The quantification of western blot results is shown in the right panel. Values are expressed as mean ± SD of three repeats. *p < 0.05; **p < 0.01; ***p < 0.001 compared to control.

To investigate whether GSP-2 could also stimulate TLR4 expression, we employed western blotting to examine TLR4 protein expression in RAW264.7 cells after stimulation by GSP-2 and LPS in the presence of PMB for 2 h or 18 h. Although no differences were observed after 2 h, TLR4 was upregulated after 18 h of treatment (Fig 3A).

Mitogen-activated protein kinases (MAPKs: ERK, JNK, and p38) are typically activated upon TLR4 engagement [25, 26]. Therefore, we examined whether these pathways were affected by GSP-2. Western blotting showed that GSP-2 activated ERK and JNK phosphorylation while phosphorylated p38 started to increase within 1h and then peaked at 8 h after GSP-2 treatment (Fig 3B).

### GSP-2 activated cytokine gene expression

**Fig 4:**
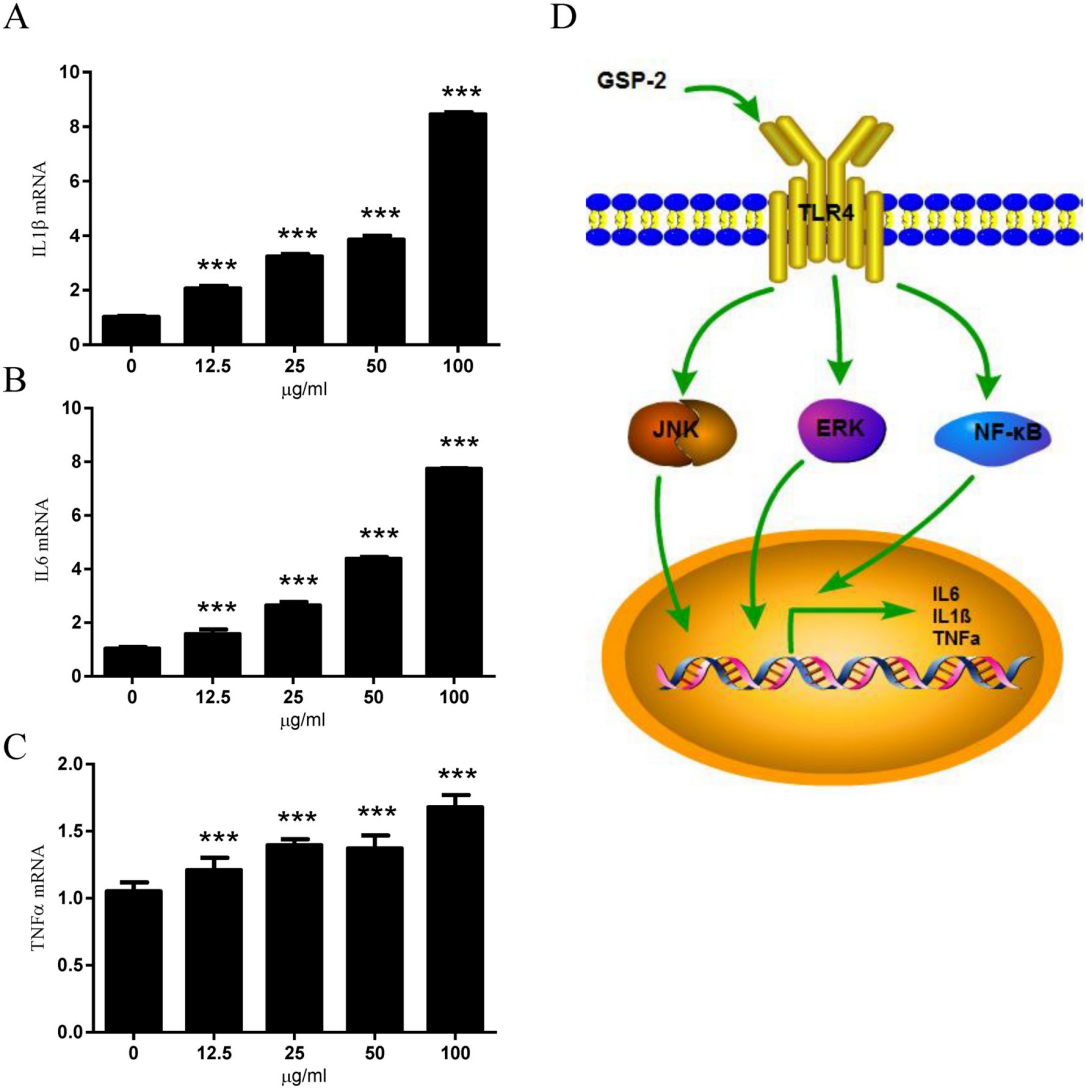
Effect of GSP-2 on the activation of cytokine genes in RAW 264.7 cells. RAW264.7 cells were incubated with the indicated GSP-2 concentrations (μg/ml) with PMB (10 μg/ml) for 18 h. The expression of IL-1β **(A)**, IL-6 **(B)**, and TNFα **(C)** genes was assessed using qRT-PCR. The gene expression level is normalized to the reference gene (GAPDH). All values are expressed as mean ± SD of three repeats. *p < 0.05; **p < 0.01; ***p < 0.001 compared to control. **(D)** Schematic illustration of the implicated molecular mechanisms.

Cytokines are not only involved in inflammation and infection responses but also have important roles in the regulation of metabolic, regenerative, and immunological processes. We investigated cytokine gene expression in RAW 264.7 cells after GSP-2 treatment for 18 h in the presence of PMB. The mRNA level of IL1β (Fig 4A), IL6 (Fig 4B), and TNFα (Fig 4C) genes were significantly upregulated after GSP-2 treatment in a dose-dependent manner. In addition, we found the similar results in monocyte-derived dendritic cells (S1 Fig). To exclude the effect of PMB, we compared the mRNA level of IL1β, IL6, and TNFα in RAW 264.7 cells with or without PMB treatment. Results showed that PMB could not upregulate expression of IL1β, IL6, and TNFα (S2 Fig). This confirmed that GSP-2 was able to activate cytokine gene expression, possibly contributing to immunity.

## Discussion

*Ganoderma Sinense* is a Chinese unique medicinal fungus that has been used in folk medicine for thousands of years [1, 4, 27]. Polysaccharides are considered biologically active ingredients due to their immune-modulating functions [28]. To obtain an in-depth characterization of the polysaccharides contained in GS, a systematic bioassay-directed extraction and isolation procedure was conducted on the dried fruiting bodies of GS. GSP-2, a water-soluble protein-bound glucan, was isolated from GS, and we have characterized its structure [7]. We have reported that GSP-2 affects immunity and induces the secretion of cytokines, but the underlying mechanism is not clear [7]. This study was the first to demonstrate that GSP-2 directly interacts with TLR4 and activates its transcription (Fig 4D).

Previously we have shown that GSP-2 can stimulate immune adaptive responses in mouse, such as proliferation of splenic B and T cells. Fluorescently labeled GSP-2 could be phagocytosed by mouse macrophages such as RAW264.7 cells [7]. In addition, GSP-2 activated nitric oxide production in RAW264.7 cells [7]. The relationship between polysaccharide origin, structure, and immunomodulatory activity needs to be further characterized.

Innate control of adaptive immunity is now a well-established paradigm. Toll-like receptors (TLRs) have a crucial role in innate immunity. They are a family of cellular receptors specifically recognizing microbes and have a primary role in innate response [29]. TLRs are thought to be the major receptors on the surface of primary immune cells and have a predominant role in the recognition of polysaccharides from this species. On the other hand, due to the complexity of polysaccharide structure, to date, the interaction between GS polysaccharides and TLRs has not been elucidated.

Therefore, in this study, a structurally characterized GS polysaccharide, GSP-2, and TLR-over expressing or -knockout cells were employed to identify the TLR member(s) implicated in GSP-2 recognition, as well as the activated downstream signaling pathways. GSP-2 dose-dependently activated TLR4 gene overexpression and upregulated TLR4 protein expression, but did not affect the expression of other TLR types. Moreover, GSP-2 was not able to induce TNFα secretion in primary macrophages from TLR4 knockout mice. These results suggested a direct interaction between GSP-2 and TLR4. Moreover, in RAW246.7 macrophages, GSP-2-induced TLR4 protein expression was associated with MAPK phosphorylation and with the transcription of downstream effectors such as PTGS2, CD14, CLEC4E, CEBPB, CCL2, CSF3, IL1β, and IL6. In addition, the pro-inflammatory transcription factor NF-κB is also activated downstream of TLR4 and induces cytokine expression. The data suggests that upregulated cytokine secretion might be partly due to activation of MAPK pathways by GSP-2. NF-κB might also serve as a potential mechanism for the downstream effects observed in addition to MAPK activation. Taken together, our experiments suggest that GSP-2 is recognized by TLR4 on the membrane of macrophages and induces the activation of signaling pathways, which might in turn lead to cytokine secretion and modulation of immunity.

Ongoing experiments will focus on various questions, including the in vivo effects of GSP-2, the optimization of GSP-2 effectiveness and safety, and the possible impact that this polysaccharide may have on additional effector pathways.

## Supporting information

S1 Table List of primers used in this work. (DOCX)

S2 Table mRNA level of 84 genes. (XLS)

S1 Fig Effect of GSP-2 on the activation of cytokine genes in monocyte-derived dendritic cells. Dendritic cells were incubated with the indicated GSP-2 concentrations (μg/ml) in the presence of PMB (10 μg/ml) for 18 h. The expression of IL-1β, IL-6, and TNFα was assessed by using qRT-PCR. The gene expression level is normalized to the reference gene (GAPDH). All values are expressed as mean ± SD of three repeats. *p < 0.05; **p < 0.01; ***p < 0.001 compared to control.
(TIFF)

S2 Fig Effect of PMB on the activation of cytokine genes in RAW 264.7 cells. RAW264.7 cells were incubated with or without PMB (10 μg/ml) for 18 h. The expression of IL-1β, IL-6, and TNFα genes was assessed using qRT-PCR. The gene expression level is normalized to the reference gene (GAPDH). All values are expressed as mean ± SD of three repeats. *p < 0.05; **p < 0.01; ***p < 0.001 compared to control.
(TIFF)
