# Supplementary material for: GSP-2, a polysaccharide extracted from Ganoderma sinense, is a novel toll-like receptor 4 agonist
Source: PLoS One. 2019 Aug 23;14(8):e0221636. doi: 10.1371/journal.pone.0221636 (PMC6707555; doi:10.1371/journal.pone.0221636)
Supplement: S1 Table — (DOCX) [file pone.0221636.s001.docx]

Primers used in this study

| Gene |  | Primer Sequence |
| --- | --- | --- |
| Mouse IL1β | Up | ACCTGGGCTGTCCTGATGAGAG |
|  | Down | TGTTGATGTGCTGCTGCGAGAT |
| Mouse IL6 | Up | TGGGACTGATGCTGGTGACAAC |
|  | Down | AGCCTCCGACTTGTGAAGTGGT |
| Mouse GAPDH | Up | AAGAAGGTGGTGAAGCAGGCATC |
|  | Down | CGAAGGTGGAAGAGTGGGAGTTG |
| Mouse TNFα | Up | TGGAACTGGCAGAAGAGGCACT |
|  | Down | AGAGGCTGAGACATAGGCACCG |
