# Supplementary figures and images for: GSP-2, a polysaccharide extracted from Ganoderma sinense, is a novel toll-like receptor 4 agonist
Source: PLoS One. 2019 Aug 23;14(8):e0221636. doi: 10.1371/journal.pone.0221636 (PMC6707555; doi:10.1371/journal.pone.0221636)

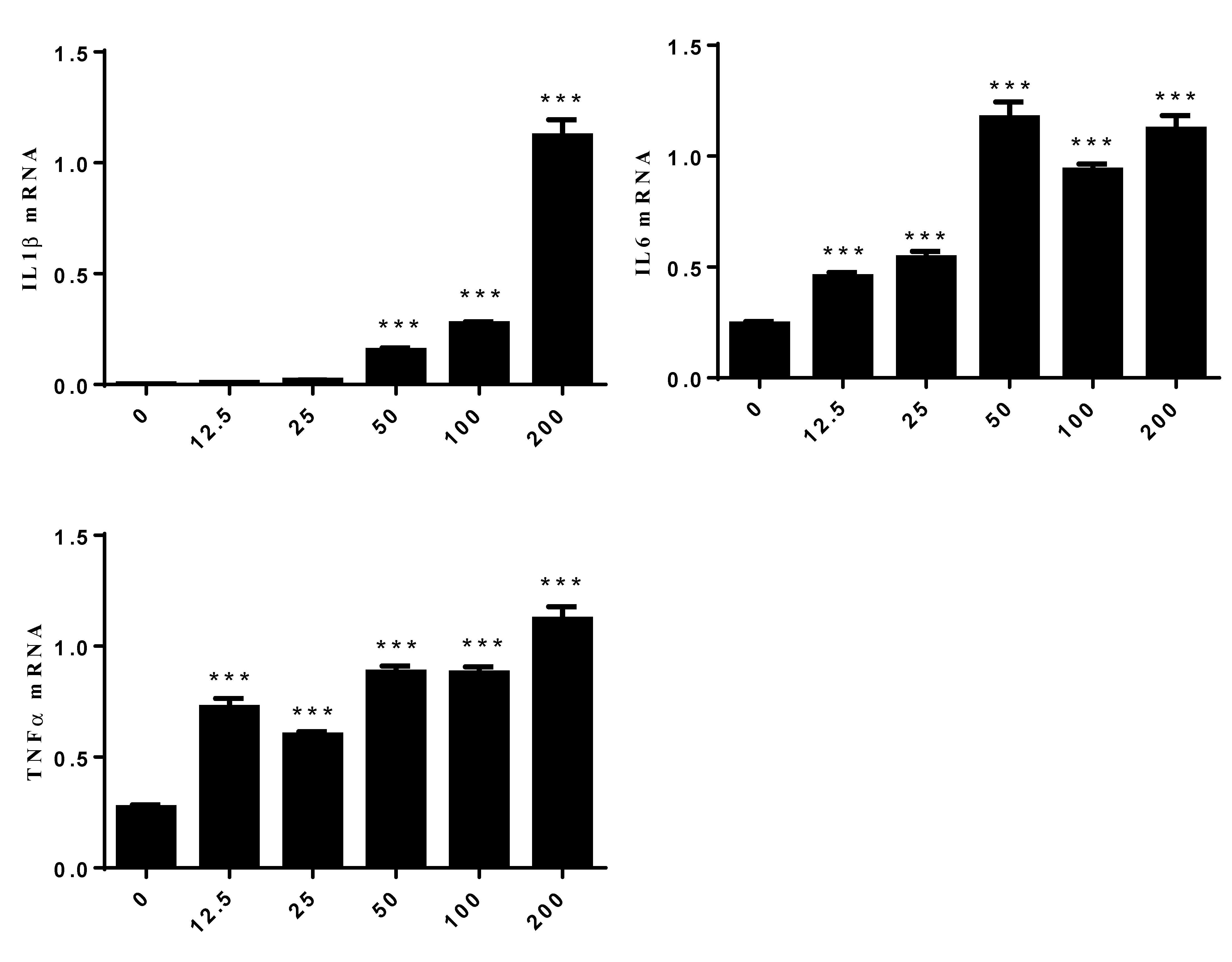

Supplement: S1 Fig — Dendritic cells were incubated with the indicated GSP-2 concentrations (μg/ml) in the presence of PMB (10 μg/ml) for 18 h. The expression of IL-1β, IL-6, and TNFα was assessed by using qRT-PCR. The gene expression level is normalized to the reference gene (GAPDH). All values are expressed as mean ± SD of three repeats. *p < 0.05; **p < 0.01; ***p < 0.001 compared to control. (TIFF) [file pone.0221636.s003.tiff]

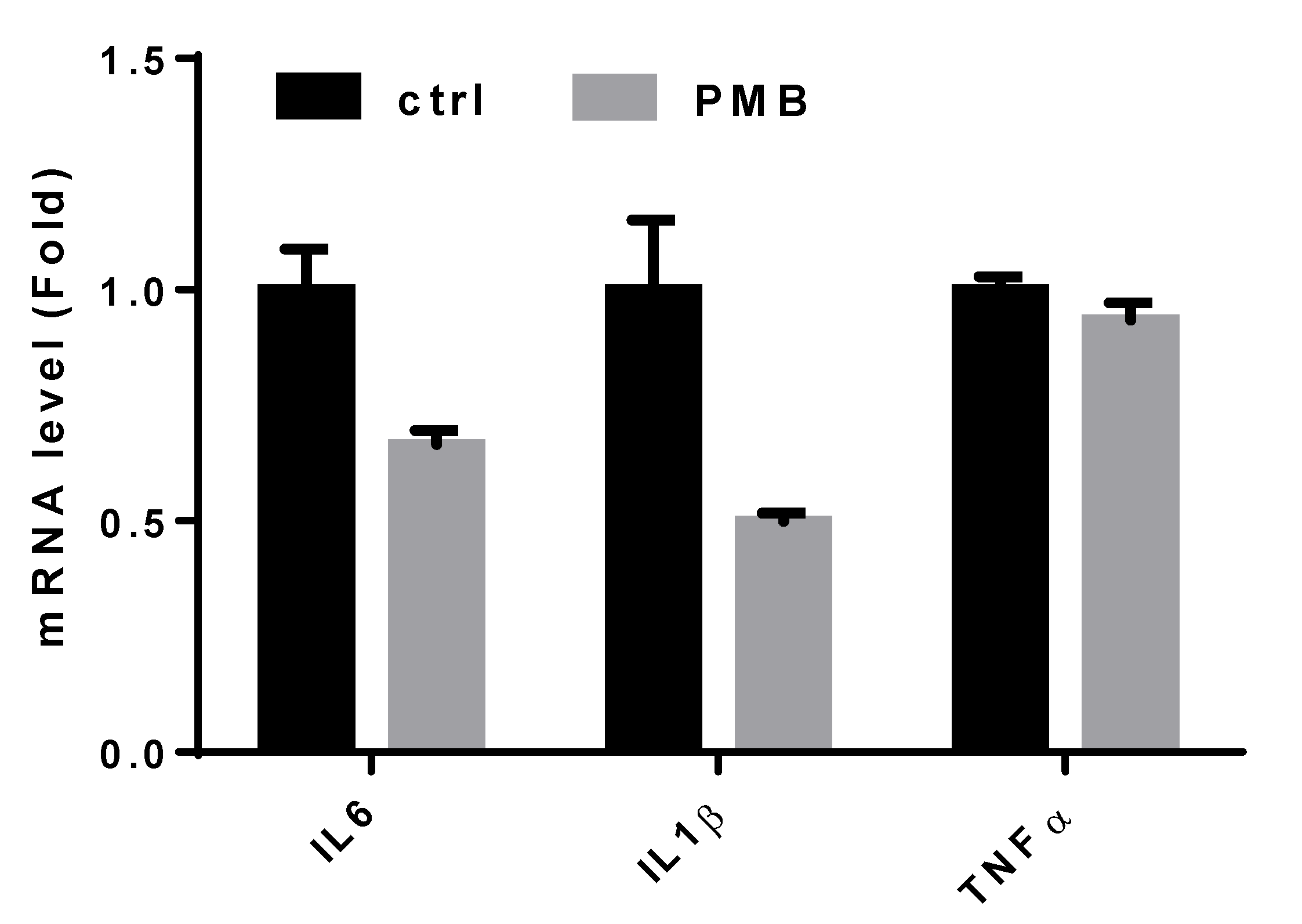

Supplement: S2 Fig — RAW264.7 cells were incubated with or without PMB (10 μg/ml) for 18 h. The expression of IL-1β, IL-6, and TNFα genes was assessed using qRT-PCR. The gene expression level is normalized to the reference gene (GAPDH). All values are expressed as mean ± SD of three repeats. *p < 0.05; **p < 0.01; ***p < 0.001 compared to control. (TIFF) [file pone.0221636.s004.tiff]
